# Composition and physico-chemical properties of bottom sediments in the southern part of the Bratsk Reservoir (Russia)

**DOI:** 10.1038/s41598-019-49228-4

**Published:** 2019-09-05

**Authors:** Mariusz Rzetala, Victoria A. Babicheva, Martyna A. Rzetala

**Affiliations:** 1 University of Silesia, Faculty of Earth Sciences, Bedzinska 60, 41-200 Sosnowiec, Poland; 2 Russian Academy of Sciences, Institute of Earth’s Crust, Lermontova 128, 664033 Irkutsk, Russia

**Keywords:** Geochemistry, Environmental impact, Hydrology, Limnology, Natural hazards

## Abstract

The paper presents the results of studies of bottom sediments taken from the southern part of the Bratsk Reservoir. The following analyses have been conducted: trace element analysis, particle-size analysis as well as chemical analysis of water, hydrochloric acid and alkaline extracts for 18 samples of the bottom sediments. The granulometric analysis has identified the predominance of fine silt and silty-clayed sediments. The data on the content of trace elements in the bottom sediments of the Bratsk Reservoir is presented in comparison with their content in the natural environment of the Baikal region; the anomaly ratio was used to characterize the excess for trace elements. The chemical analysis of extracts has showed “saline contamination” of mud (silt), high concentration of carbonates in it, as well as the presence of mobile (free) forms of aluminum oxide. In this research, an attempt was made by using a correlation analysis to evaluate the impact of various physical and chemical characteristics of the bottom sediments, such as the content of clay fraction, organic carbon, carbonates, and water-soluble salts on the accumulation of trace elements.

## Introduction

The ecological state of the entire reservoir geosystem, as well as its individual components (water mass, bio-organisms, aquatic vegetation, sediments (bottom deposits)), depends on a number of natural and anthropogenic factors. As a result of contamination, the trace element content in all components of the geosystem can vary considerably^1–5^. A dangerous trend in this case is the accumulation of toxic elements, such as heavy metals^6–17^. Most of them are biochemically active, are not subject to biodegradation and have the ability to accumulate intensively in such “comfortable” environment as bottom sediments^18,19^.

The accumulation of trace elements in bottom sediments occurs along with complex physical and chemical mechanisms of absorption, which depend on the peculiarities of the composition, structure and properties of the sediment, as well as on the properties of the absorbed compounds^20,21^. The accumulation might occur through interaction with the solid phase, following the mechanisms of ion exchange and complex formation with humic acids, adsorption by clay and other minerals, iron and manganese hydroxides, etc.^22,23^. Therefore, the accumulation and distribution of trace elements is greatly influenced by different physical and chemical characteristics of the sediment, such as granulometric composition (particle-size distribution), particularly the content of silt (clay) fraction, the content of organic carbon, carbonates, water-soluble salts, and amorphous oxides^24^. It is known that these components are involved in the formation of structural bonds of dispersive soils and, depending on the composition and amount, are able to weaken or strengthen these bonds, thus affecting the deformation and strength properties^25^. These components also have a significant effect on the physical and chemical activity of the sediments. For example, elevated concentrations of organic carbon lead to a decrease in the content of inorganic carbon and hydrogen index and, as a consequence, to the change of oxidation-reduction potential, leading to the release of metals from the sediments into the aquatic environment^26^. Due to the leaching of water-soluble compounds, the density of the texture of subsoils, their cohesive properties and permeability change. In the process of dissolution and leaching of salts, the composition of absorbed cations, which significantly affect the properties of sediments, may change^27^.

The purpose of this study, based on the results of laboratory analyses of sediments taken from the south of the Bratsk Reservoir, is to identify possible existing relationship between such physical and chemical characteristics of the bottom sediments as the content of clay fraction, organic carbon, carbonates, water-soluble salts, amorphous oxides and accumulation of trace elements.

## Study Area

The Bratsk Reservoir is the second in the cascade of three existing reservoirs on the Angara River and is the second largest (in terms of its volume −170 km^3^) in the world. The body of water is located in the south of the Central Siberian Plateau and is elongated in meridional direction, from south to north, covering the steppe, forest-steppe and taiga landscape-climatic zones. Along with the erosion-denudation and structural-denudation relief, the presence of relic accumulative and accumulation-erosion terraces (of 10–20 m and 12–80 m heights respectively) that appeared in Quaternary, can be observed. Most accumulative river terraces have the common gently-sloping surface leveled out by denudation (some terraces were flooded after creation of the Bratsk Reservoir)^28^. This reservoir is used mostly for energy purpose, which does not preclude its use for other purposes, such as for water supply, recreation, fisheries, water transport and timber floating. The Bratsk Reservoir has been operating for almost 50 years, since 1967^29^.

**Figure 1:**
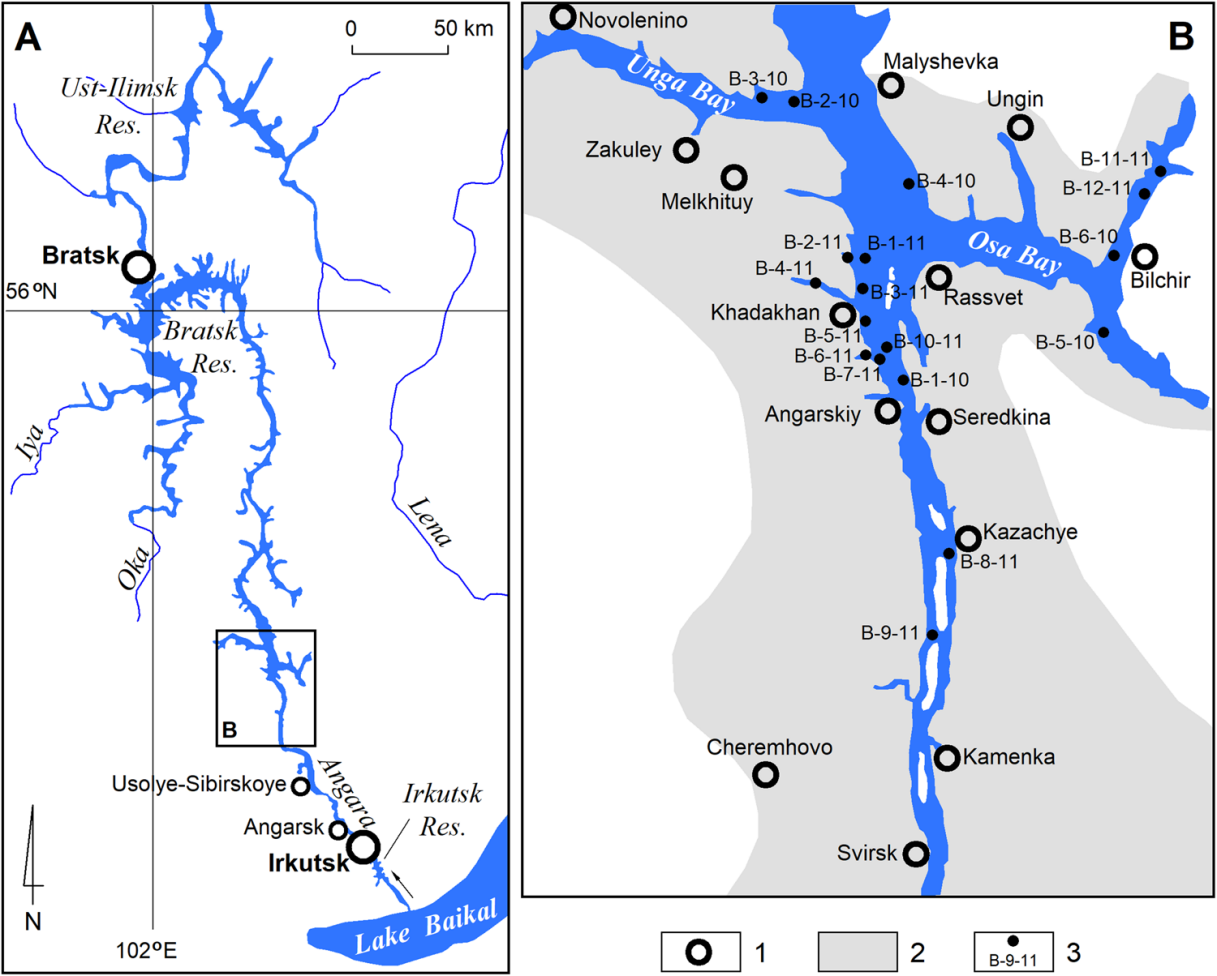
Layout of the study area: 1 – settlements; 2 – agricultural land: arable land, pastures, 3 – sediment sampling points with sample numbers.

The present exploration target is the southern part of the Bratsk Reservoir – from the transient region to the mouth of the Unginsk Creek. The width of the reservoir at the site under study varies from 1 to 7.5 km, an average of 3 km. The average depth is 9–10 m, the maximum depth is 30 m. This is the most developed part of the reservoir. There are several small towns on the shores with existing industries (Usolye-Sibirskoye – chemical industry, mechanical engineering; Svirsk – timber and woodworking industry; Cheremkhovo – coal-mining, timber and woodworking industry) and a large number of villages with well-developed agriculture^30^ (Fig. 1). The territory belongs to the forest-steppe zone strongly affected by gully erosion^31^.

The Bratsk Reservoir is fed mainly by the Angara River flowing out of Lake Baikal. The rivers flowing into the reservoir on the site under study (the Irkut, Kitoi, Belaya, Ushakovka, Kuda, Osa) are of secondary importance here. The flowage of the Bratsk Reservoir is low, on average about 2/3 of its total volume. As a result, the speed of discharge currents in the Angara river bed is 6–10 cm/sec, and in the spreading of the stream it slows down to 2–5 cm/sec^32^.

The bedrock is Lower-Middle-Cambrian rock, consisting of dolomites, limestones, gypsum, anhydrite and carbonate breccias and Middle-Upper-Cambrian rock, which consist of marl, dolomite, argillites and aleurolite intercalations with thin gypsum lenses. The alluvial deposits are represented by sands – from fine- to medium-grained, with interbedded clay and loam, fine sandy loams, loams and gravels. The slopes and alluvium of river terraces are covered with deluvial (talus) clay loam and fine sandy loam of small thickness – 10–15 m^33^.

## Sampling and Analysis

Sediment samples were collected from 15 different locations of Bratsk Reservoir in 2010 and 2011 using a sediment core sampler operated from a boat. The sampler was submerged on a steel line using a hand-operated winch. In total 18 samples from a sediment layer of a thickness of about 10 cm were collected. Sampling depth varied from 2.5 m to 28.0 m.

The selected samples were dried to air-dry state *in vitro*, and then each sample was divided into 2 parts. After the homogenization process, one part was packed in a plastic bag and sent to ACTLABS (Activation Laboratories Ltd.) in Canada to be analyzed for the trace element contents. Laboratory analyses in 2010 were conducted using TD-ICP (total digestion-inductively coupled plasma) method with respect to Cu, Mo, Ni, Pb and Zn. A 0.25 g sample aliquot is digested with HClO4–HNO3–HCl–HF at 200 °C to fuming and is then diluted with aqua regia^34,35^. This leach is partial for magnetite, chromite, barite and other spinels and potentially massive sulphides. The other trace element contents were determined using the INAA (instrumental neutron activation analysis) method. A 1 g aliquot is encapsulated in a polyethylene vial and irradiated with flux wires and an internal standard (1 for 11 samples) at a thermal neutron flux of 7 × 10^12^ n cm^−2^ s^−1^. After a 7-day decay to allow Na-24 to decay the samples are counted on a high purity Ge detector with resolution of better than 1.7 keV for the 1332 keV Co-60 photopeak. Analysis results are given in ppm^34,35^.

In 2011 analyses were conducted using the Aqua Regia – ICP/MS method. A 0.5 g sample is digested in aqua regia at 90 °C in a microprocessor controlled digestion block for 2 hours. Digested samples are diluted and analyzed by Perkin Elmer Sciex ELAN 6000, 6100 or 9000 ICP/MS^34,35^.

Another part of each sample was analyzed in the Analytical Centre of the Institute of the Earth Crust (Irkutsk, Russia) using a special method developed for analysing clay and loessial soils^25^. Clay and loess deposits are complex natural formations consisting of the primary particles and aggregates. The presence of aggregates affects the results of particle size (granulometric) analysis, as it reduces the content of fine particles and increases the content of coarse ones. This distorts the correct understanding of the massive material, the content of certain particles in it and its classification. The particle size analysis shows the limiting (extreme) dispersion, but in order to obtain information about the natural dispersion, it is necessary to know the number of secondary micro-aggregate particles. This method allows obtaining such information without isolating the micro-aggregate composition or performing additional micro-aggregate analysis of the soil. Namely, the granulometric analysis was made by pipette method, using 3 processes of specimen preparation^35^: (1) aggregate process (shaking up of the specimen in water causes the destruction of coarse water-unstable aggregates); (2) the semi-disperse process (boiling of triturated specimen in presence of ammonia), and (3) disperse preparation (boiling of triturated specimen with sodium pyrophosphate leads to maximum destruction of aggregates). The semi-dispersed technique is a standard one and it is recommended for determining the particle size distribution of clay soils and for naming the sample within the framework of the classification.

By water, muriatic and alkaline extracts the components and structural bonds such as water soluble salts, carbonates, amorphous sesqui-oxides and mobile Al_2_O_3_ forms were determined. To a certain extent, water extract imitates the material composition of the soil solution, so it can help get an insight into the content and composition of the most mobile soil components that are more capable of migration. The principle of the water extraction method is the short-term (3 min.) interaction of a soil sample with distilled water, at the ratio 1:5. At the same time, the dissolution of readily-soluble salts occurs, while medium- and low-soluble salts are partially dissolve. Analysis of the extract begins with determining the carbonate and total alkalinity, as well as the pH of the extract, then the amount of dry residue is determined (the content of water-soluble salts is determined as the sum of anions and cations) after evaporation in a water bath. When analysing the hydrochloric acid extract, the contents of R_2_O_3_, SiO_2_, CaO, MgO, Fe_2_O_3_, SO_3_ as well as the loss on ignition and the value of the mineral insoluble residue are obtained. Then, using special coefficients, oxides are recalculated for the content of carbonate salts (CaCO_3_, MgCO_3_, FeCO_3_) and their sum is determined, which characterizes the total carbonate content of the soil. Analysis of the alkaline extract allows us to determine the content of mobile Al_2_O_3_ forms^25,36^.

The humus content was determined according to I.V. Tyurin’s method based on the oxidation of carbon of organic matter by excess potassium bichromate. The oxidation occurs in a highly acidic medium and is accompanied by the reduction of hexavalent chromium to the trivalent. Excess bichromate in the solution after oxidation of organic matter is titrated with a solution of Mohr’s salt. Based on the difference of potassium bichromate – before and after oxidation – the content of organic carbon in the soil is found^37^.

## Results and Discussion

The material composition of the bottom sediments at the site under study refers to the terrigenous genetic type. The terrigene sediments are formed from detrital or pelitic (lutaceous) material, originating from the banks, due to various exogenous processes^38^. As a result of mechanical differentiation under the action of waves, currents, underwater gravitational processes, etc., coarse deposits usually prevail in the area of submerged shoals: pebbles, gravel, and sand. With distance from the shoreline, they are replaced by fine sands and silts.

### Granulometric analysis (particle-size distribution) of bottom sediments

**Figure 2:**
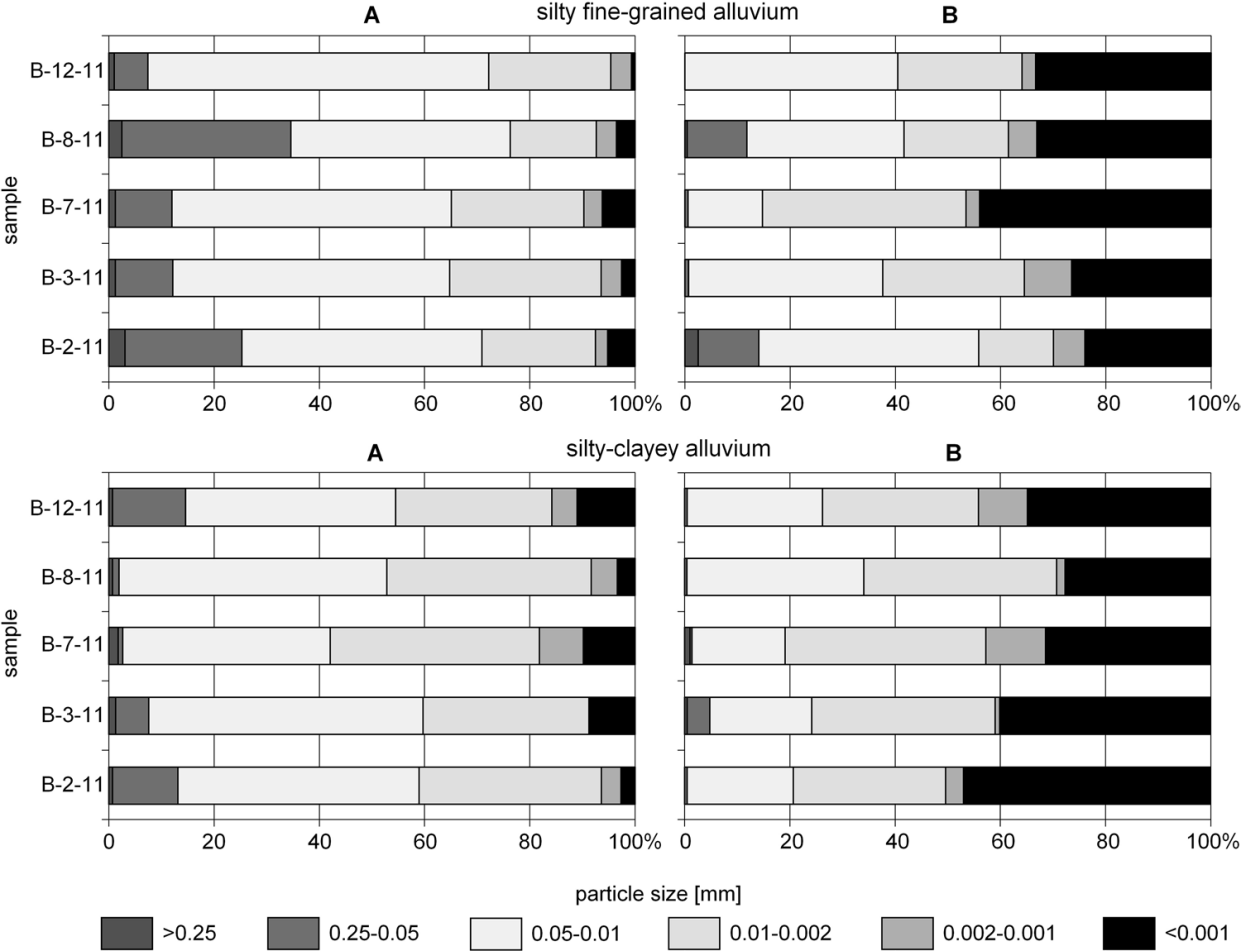
The particle contents in the Bratsk Reservoir sediment samples: (**A**) – semi-dispersed specimen preparation, (**B**) – dispersed specimen preparation.

The granulometric analysis of the sediments from the south of the Bratsk Reservoir has determioned the predominance of fine silt and silty-clayed muds. The sediment received its name according to the particle-size distribution of the sample with semi-dispersed preparation. Figure 2 shows a batch of the ten most representative samples from the groups of finely-aleuritic (B-12-11, B-8-11, B-7-11, B-3-11, B-2-11) and aleuritic-clayed (B-11-11, B-6-11, B-5-11, B-4-11, B-5-10) silt^35^. The particle-size distribution of each sample is presented in two versions of sample preparation: a – semi-disperse, and b – dispersed ones. The graphs demonstrate how the ratio of particle contents by fractions significantly changes towards the increased number of more micro-fine particles with maximum destruction of aggregates in the case of dispersed sample preparation.

In fine silt muds, the content of 0.05–0.01 mm fraction is mainly up to 50–70% (Fig. 2). The fractions of 0.25–0.05 mm and 0.010–0.002 mm have approximately the same ratio. The total content of silt with particle size of <0.01 mm does not exceed 40%. Aleuritic-clayey silts generally contain no more than 50% of 0.05–0.01 mm particle size (but when the dispersed sample preparation is used, their content decreases sharply several times in favor of fine-grained particles <0.001 mm in size), and more than 40% of clay particles <0.01 mm in size. The content of the sand fraction (>0.25 mm) in almost all samples does not exceed 1%.

### Trace element composition of bottom sediments

The average, minimum and maximum trace element contents in the sediments from the south of the Bratsk Reservoir are presented in Table 1^22,35,38–42^. As a rule, environmental assessment of the levels of content of chemical elements in the natural environments is carried out by comparing their actual concentrations versus regulatory (standardized) parameters. However, to date there are no regulatory requirements for the maximum permissible trace element concentration for the sediments in Russia. Therefore, in order to assess the quality of bottom sediments in the Bratsk Reservoir, Table 1 shows the values of percentage abundance (clarks) of trace elements in the earth’s crust, according to Rudnick and Gao^42^, as well as the average trace element contents in the bedrock of the Baikal region^22,39^ and abyssal silt of Lake Baikal^40,41^ (for certain elements). The studies show that for such trace elements as Co, Cr, Cu, Mn, Ni, Pb, Th, V, Zn, the exceeding in the geochemical background of bedrock in the region is observed. It may be noted that the average trace element contents in the deep (abyssal) silt of Lake Baikal for the most part can be checked against the maximum contents in the bottom sediments of the Bratsk Reservoir or exceed them.

**Table 1:** Trace element contents in the bottom sediments of the Bratsk Reservoir, versus their content in the natural environment of the Baikal region, as well as calculated anomaly ratio (AR).

Element	Content [ppm]			Percentage abundance in the Earth’s crust^42^ [ppm]	Average content in the natural environment of the Baikal region [ppm]		AR
	average	max	min		bedrock^22,39^	deep (abyssal) silt of Lake Baikal^40,41^	
Be	1.30	2.00	0.80	2.10	2.0	—	0.63
Co	18.10	28.00	12.20	17.30	9.0	19.00	2.01
Cr	95.30	176.00	45.10	92.00	80.0	120.00	1.19
Cs	2.70	5.00	1.11	4.90	6.2	5.00	0.44
Cu	31.30	44.00	15.70	28.00	29.0	—	1.08
La	28.30	41.90	14.30	31.00	35.2	58.00	0.80
Mn	717.60	1200.00	424.00	1000.00	685.0	945.00	1.05
Mo	0.80	2.09	0.35	1.10	8.3	1.40	0.10
Ni	68.00	97.00	45.30	47.00	32.0	84.00	2.12
Pb	13.80	19.00	8.18	17.00	11.0	19.00	1.25
Rb	42.60	100.00	16.00	84.00	42.80	80.00	1.00
Sc	10.70	20.30	4.70	14.00	11.00	13.00	0.97
Sr	140.40	250.00	45.00	320.00	210.0	320.00	0.67
Th	5.70	8.90	1.20	10.50	3.8	10.00	1.49
V	83.90	138.00	49.00	97.00	62.0	160.00	1.35
Y	18.00	29.00	11.50	21.00	22.5	–	0.8
Zn	89.50	108.00	62.80	67.00	41.0	88.00	2.18

Explanation: (−) – data is not available.

Thus, in order to determine the excesses of the aforementioned trace elements (Co, Cr, Cu, Mn, Ni, Pb, Th, V, Zn), anomaly ratio (AR) was used, which is calculated as the ratio of the content of a chemical element in the studied natural object to the geochemical background^43^. So, the highest excess of the background (among the aforementioned) is recorded for Co, Ni and Zn (AR is 2.0–2.18). The average content of other elements exceeds the geochemical background less than 1.5 times.

### Structure-forming components

**Figure 3:**
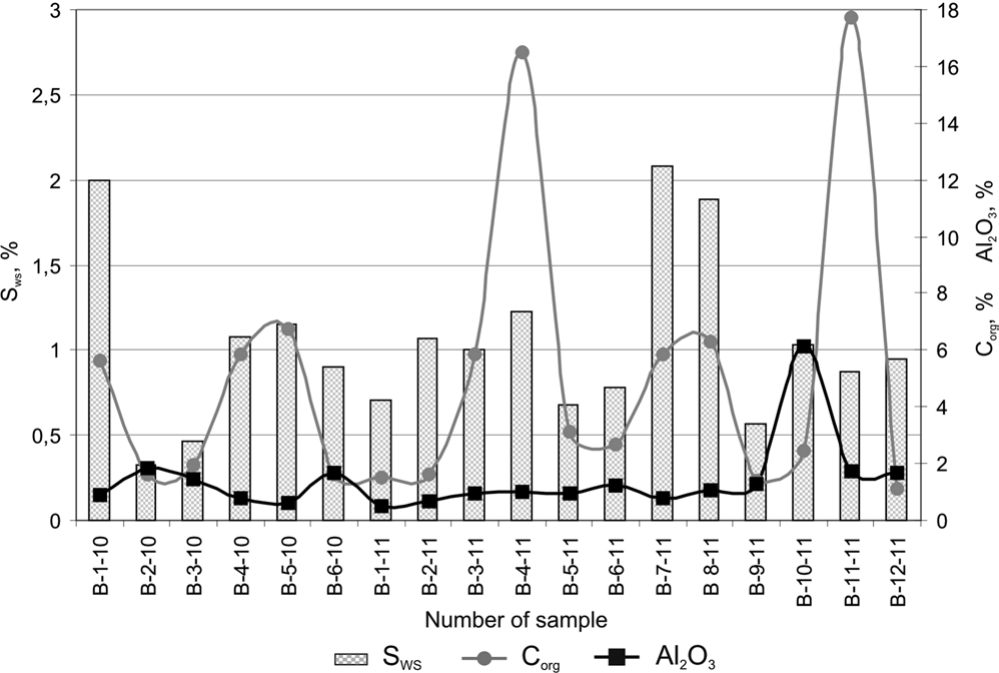
Total content of water-soluble salts (S_ws_), humus (С_org_) and mobile forms of aluminum oxide (Al_2_O_3_).

According to the results of chemical analysis of aqueous extract, silts in the Bratsk Reservoir are characterized by varying degrees of salinity: from medium saline (Water-soluble salt contents, S_ws_ % range within 0.329–0.944%)^35^ – to extremely highly saline (S_ws_ is up to 2.080%); the average value is 1.042% (Fig. 3). The salinity quality is predominantly of carbonate-sulfate type; it is caused by a high degree of water solubility of the chloride salts, which practically do not accumulate in the sediments.

**Figure 4:**
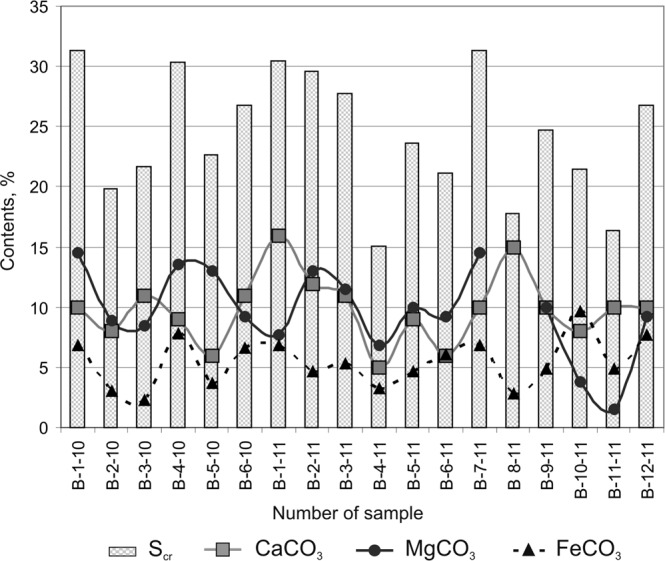
Composition of carbonate salts, total content of carbonates (S_cr_).

The total content of carbonates in the sediments varies widely, from 15.08 to 31.32%^35^, on average it is 24.36% (Fig. 4). CaCO_3_ and MgCO_3_ as prevail in the content of carbonate salts. According to Ryashchenko^38^, the constant presence of CaCO_3_ dominated carbonates is a regional feature of quaternary sediments (loessial and clayey soils) in the south of Eastern Siberia, as well as salinity, however, at a weaker level (S_ws_ varies within 0.06–0.42%) and predominantly of chloride-carbonate type. The so-called “saline contamination” of sediments in the Bratsk Reservoir might be technology-related and can be linked to the close proximity of industrial areas.

The content of mobile (free) forms of aluminum oxide (Al_2_O_3_), which plays the role of cement taking part in the formation of the sediment’s structural connections and contributing to the increased particle aggregation, is 0.48–6.13% (an average of 1.43%) (Fig. 3).

The humus content ranges within 1.14–17.70%, an average of 5.60% (Fig. 3). Based on the studies by Butakov^44^, it was determined that the bulk organic carbon in the sediments of the Bratsk Reservoir accounts for humic acid. The proportion of organic carbon (С_org_) in humic acids (both bound and free, in total) reaches 62%. This indicates a high degree of humification of the reservoir bottom sediments, which, in turn, suggests the allochthonous nature of the humic substance in the reservoir sediments.

### Influence of physical and chemical characteristics of bottom sediments on the distribution of trace elements

Table 2 presents Pearson’s correlation coefficients between the contents of Co, Cr, Cu, Mn, Ni, Pb, Th, V, Zn (trace elements for which the excess of geochemical background of bedrock in the region was marked), such physical and chemical parameters as the content of pelitic (clayey) particles (<0.01 mm) in a sample with different variants of sample preparation, as well as the content of calcium and magnesium carbonates, organic carbon and water soluble salts.

**Table 2:** Correlation matrix of the content of heavy metals in bottom sediments of the Bratsk Reservoir.

Characteristic	Agg	SD	D	Co	Cr	Cu	Mn	Ni	Pb	Th	V	Zn	CaCO_3_	MgCO_3_	С_org_
SD	0.850														
D	0.648	0.652													
Co	0.823	0.588	0.510												
Cr	0.598	0.420	0.417	0.828											
Cu	0.658	0.487	0.495	0.886	0.962										
Mn	0.287	0.593	0.442	0.093	0.003	−0.054									
Ni	0.849	0.561	0.567	0.956	0.873	0.887	0.071								
Pb	0.597	0.630	0.562	0.651	0.783	0.806	0.258	0.645							
Th	0.301	0.045	0.006	0.524	0.706	0.675	−0.402	0.556	0.434						
V	0.058	0.199	−0.039	0.386	0.479	0.501	0.065	0.261	0.406	0.066					
Zn	0.629	0.494	0.479	0.865	0.933	0.959	−0.001	0.855	0.776	0.505	0.608				
CaCO_3_	−0.654	−0.537	−0.400	−0.452	−0.409	−0.405	−0.021	−0.524	−0.456	−0.065	−0.156	−0.490			
MgCO_3_	−0.065	−0.067	0.046	0.282	0.442	0.414	−0.079	0.210	0.320	0.518	0.473	0.325	0.058		
С_org_	0.194	0.288	0.459	0.033	−0.246	−0.094	0.223	−0.026	0.089	−0.686	0.022	0.058	−0.313	−0.497	
S_ws_	−0.210	−0.396	0.178	−0.042	−0.261	−0.151	−0.334	−0.110	−0.304	−0.022	−0.334	−0.202	0.163	0.203	0.217

Explanations:

Agg – the content of pelitic particles (<0.01 mm) in samples with aggregate preparation;

SD – the same, with semi-dispersed preparation;

D – the same, with dispersed preparation.

Many studies have confirmed that particle-size distribution of sediments affects the transport and accumulation of high-density (heavy) metals in soils^45,46^. Huang *et al*. have found that Cu, Ni and Mn showed a positive correlation with clay proportions because clay particles are more prone to adsorb heavy metals^47^. In this study, the correlation analysis has also showed that the highest value in the accumulation of trace elements in the sediments is their dispersion, i.e., the content of particles <0.01 mm in size. Co, Cr, Cu, Ni, Pb and Zn demonstrated the most significant positive correlation in terms of the content of pelitic (clayey) particles in an aggregated state (r ranges from 0.597 (Pb) to 0.849 (Ni)). When semi-dispersed and dispersed sample preparation is used, i.e., when aggregates are destroyed, this correlation is weakened for most elements, as indicated by a decrease in the correlation coefficient. Thus, when semi-dispersed and dispersed sample preparation is used, only Co, Ni and Pb show the average degree of correlation (0.5 < r < 0.69) with the content of pelitic particles, while Cr, Cu and Zn show the moderate correlation (0.3 < r < 0.49).

The influence of organic matter on the accumulation of heavy metals in the sediments is insignificant. This is indicated by the correlation coefficients of the content of organic carbon and heavy metals in the sediments, varying from −0.313 to 0.459 (the exception is an inverse correlation of С_org_ with Th (r = −0.686)). The weak correlation between concentrations of heavy metals in the sediments containing organic matter in most cases suggests the secondary role of humic substance in the process of accumulation and migration of metals, and it is the evidence of predominantly terrigenous nature of the formation of trace-element composition of the bottom sediments in the Bratsk Reservoir.

The territory under study is intensively used for agricultural purposes. Farms tend to use large quantity of fertilizers (both mineral and organic ones) and pesticides to promote the acceleration of agricultural production. As numerous studies show, this practice often leads to the accumulation of trace such elements as Cr, Ni, Cu, Zn, As, Cd and Pb in soils^45,48,49^. Soil erosion, being an important factor in the redistribution of soil in the reservoir, is probably the main mechanism by which the studied trace elements enter the bottom sediments of the reservoir^50^.

The correlation analysis has showed a average negative correlation of the content of calcium carbonate CaCO_3_ with Ni (r = −0.524), as well as a moderate negative correlation with such trace elements as Co, Cr, Cu, Pb and Zn. The content of magnesium carbonate MgCO_3_ moderately positively correlates with such trace elements as Cr, Cu, Pb, V and Zn. Besides, there is a moderate negative correlation between the content of water soluble salts and S_ws_ and the content of Mn, Pb and V in the bottom sediments. Interestingly, metals are correlated with each other. A particularly strong positive correlation (r > 0.70) is observed between Co, Cr, Cu, Ni, Pb and Zn. Such a pronounced correlation might indicate that these trace elements have the same source of influent into the reservoir^51^. Mn is the least active in correlation with other trace elements (its correlation is generally very weak, r < 0.19). For Th and V, the correlation with other trace elements mainly varies from very low to moderate.

Thus, the correlation relations confirm the dependencies – both well-known and specific for the reservoir under study – between the analysed features of the bottom sediments. First of all, it is the relationship between particle size and the content of toxic metals, which can be attributed to the conditions of sedimentation of sediments in the reservoir. The accumulation of trace elements in finely dispersed sediments can be viewed as a manifestation of the purifying role, especially in the zone of a variable backwater of the reservoir, but also as an increasing threat of environmental pollution. Significant correlation coefficients of the content of most toxic trace elements in the samples under study suggest the complex nature of the influent of pollutants into bottom deposits.

## Conclusions

1. Based on the results of the granulometric analysis, the sediments in the south of the Bratsk Reservoir are mainly represented by fine silt and silty-clayed muds with a high degree of fine particles aggregation.
2. The trace element composition of the bottom sediments includes a number of elements, the content of which exceeds the values of the geochemical background of the bedrock in the region: Co, Cr, Cu, Mn, Ni, Pb, Th, V, and Zn. Among these, the highest excess of the background is recorded for Co, Ni and Zn (AR 2.0–2.18), the average contents of other elements exceed the geochemical background less than 1.5 times.
3. According to the results of chemical analysis of extracts, the muds (silts) are characterized by various degrees of “saline contamination”, high concentration of carbonates, and the presence of mobile (free) forms of aluminum oxide. The wide ranges of variation of these indicators suggest technogenic pollution of certain water lots in the reservoir area.
4. The content of organic carbon in the samples also varies widely, and in some cases it reaches high values (up to 17.70%). The high degree of humification of the bottom sediments might indicate an external source of organic matter entering the reservoir. As shown by the correlation analysis, humic substance has the secondary role in the process of accumulation and migration of metals, and it is the evidence of predominantly terrigenous nature of the formation of trace-element composition of the bottom sediments in the Bratsk Reservoir.
5. The content of heavy metals in the reservoir sediments is controlled mainly through the dispersion of bottom deposits – the content of pelitic (lutaceous) particles (<0.01 mm) in the aggregated state. No significant correlation between the content of carbonates and water-soluble salts and the accumulation of trace elements under consideration has been identified. There is a strong direct relationship between many heavy metals, which indicates the same source of their influent into the reservoir.
